# Complete Genome Sequence of Feline Panleukopenia Virus Strain HRB-CS1, Isolated from a Domestic Cat in Northeastern China

**DOI:** 10.1128/genomeA.01556-14

**Published:** 2015-03-26

**Authors:** Chunguo Liu, Yongxiang Liu, Dafei Liu, Zheng Qiu, Jin Tian, Dongchun Guo, Zhijie Li, Ming Liu, Yijing Li, Liandong Qu

**Affiliations:** a State Key Laboratory of Veterinary Biotechnology, Harbin Veterinary Research Institute, Chinese Academy of Agricultural Sciences, Harbin, Heilongjiang, China; b College of Veterinary Medicine, Northeast of Agricultural University, Harbin, Heilongjiang, China

## Abstract

Here, we report the complete genome sequence of feline panleukopenia virus (FPLV) strain HRB-CS1, isolated from a dead domestic cat showing enteric symptoms in China in 2014. The genome of HRB-CS1 was sequenced and analyzed, which will help to understand the genetic characteristics and evolution of FPLV in China.

## GENOME ANNOUNCEMENT

Feline panleukopenia virus (FPLV) is a member of the genus *Parvovirus*, in the family *Parvoviridae*. FPLV is also an important viral pathogen of cats that can cause acute gastroenteritis and leucopenia and is sometimes fatal for kittens (1). FPLV can infect many felids, including cats, tigers, and lions (2). Recently, FPLV isolated from a monkey caused a severe outbreak of hemorrhagic enteritis in young monkeys with a mortality rate of 50% (3), which suggested that FPLV has the potential to infect other mammals. Therefore, the whole-genome analysis is very important for discovering the mechanism of genetic evolution and the pathogenicity of FPLV.

In this study, FPLV strain HRB-CS1 was isolated from a dead domestic cat with enteritis in Heilongjiang province, northeastern China, in 2014, and the full-length genome was sequenced and analyzed. The samples were treated and inoculated with a Crandell-Rees feline kidney (CRFK) cell monolayer as previously described (4), and the virus was purified by plaque assay. The viral DNA was extracted by the Body Fluid Viral DNA/RNA mini kit (Axygen). The 2.1-kb, 1.6-kb, and 1.6-kb overlap DNA fragments covering the genome were amplified by LA Taq DNA polymerase (TaKaRa) with three specific primers and were cloned into the pMD18-T vector; the positive clones were sequenced by FPLV specific primers and vector primers by the Boshi Biotechnology Company.

The nearly full-length genome of strain HRB-CS1 comprises 4,688 nucleotides (nt). The bioinformatic analysis showed that the isolate encoded four proteins, including two nonstructural proteins of NS1 (nt 114 to 2120) and NS2 (nt 114 to 373 and 1846 to 2083), and two structural proteins of VP1 (nt 2127 to 2158 and 2223 to 4382) and VP2 (nt 2628 to 4382) through alternative splicing. The homologous analysis of the nucleotide sequence of HRB-CS1 indicated that the genome showed the highest identity (99.2%) with FPV strain 193/70, that the NS1 gene was closely related to CPV-N, CPV-b, and FPLV XJ-1 (99.3%), and that the VP2 gene shared the highest homology to FPLV HT-69 (99.8%). Host-specific sites in the VP2 gene of HRB-CS1 were identical to those of most FPLV reference strains. Phylogenetic analysis of the nucleotide sequence of HRB-CS1 showed that the whole genome and the NS1 gene were closely related to strain XJ-1 and that the VP2 gene was located in the PFLV branch originating in tigers and clustered in the FPLV branch. The analysis of recombinant detection program GARD discovered that FPLV strain HRB-CS1 was a recombinant virus between FPLV and CPV and was the same as the China isolate strain FPLV XJ-1 (1).

Furthermore, compared with the CPV-b strain, the genome of HRB-CS1 had a 60-nt insertion in the 3′ untranslated region (UTR), which was similar to FPLV 193/70, FPLV CU-4, and MEV Abashiri. However, some other CPV and MEV strains with a 3′ UTR available in GenBank kept a different pattern of deletion. The function was not clear, and further study should be carried out.

### Nucleotide sequence accession number.

The genome sequence of FPLV strain HRB-CS1 has been deposited in GenBank under the accession number KP280068.
